# A Variabilidade da Frequência Cardíaca em Repouso está Independentemente Associada aos Escores de Classificação de Gordura Visceral em Homens Adultos Sauditas

**DOI:** 10.36660/abc.20220780

**Published:** 2024-05-07

**Authors:** Syed Shahid Habib, Shaea Alkahtani, Nouf Aljawini, Syed Mohammad Habib, Andrew A. Flatt

**Affiliations:** 1 King Saud University College of Medicine Department of Physiology Riyadh Arábia Saudita Department of Physiology, College of Medicine, King Saud University, Riyadh – Arábia Saudita; 2 King Saud University College of Sport Sciences and Physical Activity Department of Exercise Physiology Riyadh Arábia Saudita Department of Exercise Physiology, College of Sport Sciences and Physical Activity, King Saud University, Riyadh – Arábia Saudita; 3 King Saud University College of Applied Medical Sciences Department of Community Health Sciences Riyadh Arábia Saudita Department of Community Health Sciences, College of Applied Medical Sciences, King Saud University, Riyadh – Arábia Saudita; 4 Sulaiman Al Rajhi Colleges College of Medicine Al Bukairiyah Arábia Saudita College of Medicine, Sulaiman Al Rajhi Colleges, Al Bukairiyah – Arábia Saudita; 5 Georgia Southern University-Armstrong Biodynamics and Human Performance Center Department of Health Sciences and Kinesiology Savannah EUA Department of Health Sciences and Kinesiology, Biodynamics and Human Performance Center, Georgia Southern University-Armstrong, Savannah – EUA

**Keywords:** Gordura Intra-Abdominal, Frequência Cardíaca, Sistema Nervoso Autônomo

## Abstract

**Fundamento:**

O tecido adiposo visceral (TAV) pode ser um contribuinte modificável específico para o comprometimento autonômico relacionado à composição corporal.

**Objetivos:**

Comparar a variabilidade da frequência cardíaca (VFC) entre grupos estratificados pela classificação de gordura visceral (CGC) e comparar associações entre VFC e métricas de composição corporal.

**Métodos:**

Um estudo transversal foi realizado em homens saudáveis (n=99, idade=37,8±13,4 anos, índice de massa corporal [IMC]=26,9±4,6 kg/m^2^). A VFC foi derivada de registros eletrocardiográficos de 5 minutos. A composição corporal (percentual de gordura corporal, CGC e relação entre massa muscular e gordura visceral [RMMCGC]) foi estimada por meio de análise de impedância bioelétrica tetrapolar. Os participantes foram categorizados em grupos de acordo com a CGC: G1 (CGC=1-8); G2 (CGC=9-12); e G3 (CGC>12). Comparações ajustadas por idade foram feitas entre os grupos. Associações independentes foram quantificadas com regressões lineares múltiplas. P <0,05 foi significativo.

**Resultados:**

Raiz quadrada média de diferenças sucessivas (RMSSD) e desvio padrão dos intervalos RR normais (SDNN) foram maiores para G1 vs. G2 e G3 (p<0,05). A potência de baixa frequência (BF) foi maior no G1 que no G2 (p<0,05). CGC e RMMCGC foram associados negativamente com SDNN, RMSSD, BF e AF (p<0,05). Depois de ajustar para idade, IMC e pressão arterial sistólica e diastólica, a CGC foi significativamente preditiva de RMSSD, SDNN e AF (p = 0,002, −0,027), e a RMMCGC foi significativamente preditiva de RMSSD e SDNN (p = 0,020, −0,023).

**Conclusões:**

Os homens na categoria de CGC mais baixa tiveram a VFC mais alta. A CGC foi mais fortemente associada à VFC do que ao percentual de gordura corporal e à RMMCGC. Os parâmetros no domínio do tempo foram mais sensíveis ao TAV do que os parâmetros no domínio da frequência. Os parâmetros da VFC podem ser os principais parâmetros de interesse no rastreamento do estado autonômico cardíaco em resposta a intervenções que visam a redução do TAV.

## Introdução

As doenças cardiovasculares (DCV) continuam a ser um fardo global para a saúde, sendo uma das principais causas de morbilidade e mortalidade.^1^ Um crescente conjunto de evidências demonstra uma associação entre aumento da adiposidade e desequilíbrio do sistema nervoso autônomo (SNA),^2 - 4^ inferido a partir da análise da variabilidade da frequência cardíaca (VFC). A VFC reflete o controle cardíaco mediado centralmente via inervação autonômica.^5^ A influência vagal aumenta a VFC, enquanto o funcionamento vagal atenuado, ou o aumento da estimulação simpática, reduz a VFC.^6^ A hipoatividade cardíaco-parassimpática está associada a um risco elevado de doença coronariana incidente e mortalidade por todas as causas.^7^ Contudo, a VFC é modificável com a mudança do estilo de vida e parece ser sensível a mudanças na composição corporal.^8^

Embora a massa corporal elevada e a massa gorda tenham sido associadas à redução da VFC em uma variedade de populações jovens,^9^ saudáveis,^10^ atléticas^11^ e clínicas,^12^ a deposição de gordura específica do local não é contabilizada nesses marcadores gerais de composição corporal. O tecido adiposo visceral (TAV) que envolve órgãos vitais no abdômen é mais perigoso para a saúde humana do que a gordura subcutânea e é mais preditivo de risco cardiometabólico.^13^ Entre os indivíduos obesos, aqueles com menos armazenamento de TAV apresentam normalmente um perfil de risco mais favorável do que aqueles com TAV mais elevado. Um número limitado de investigações comparou associações entre VFC e marcadores de gordura corporal total e armazenamento central de gordura. Estudos em mulheres, idosos e população coreana relataram associações mais fortes entre VFC e indicadores de TAV.^14 - 16^ Assim, a adiposidade central parece estar influenciando a associação entre gordura corporal e função autonômica cardíaca.

O TAV pode ser estimado com análise de impedância bioelétrica tetrapolar (BIA), que serve como uma alternativa conveniente às medidas laboratoriais de critério ou à antropometria básica.^17^ Até onde sabemos, apenas uma investigação anterior quantificou associações entre TAV derivado de BIA e VFC em adultos saudáveis. Os resultados mostraram menor VFC mediada por vagal em estudantes da área da saúde com excesso de peso em comparação com um grupo de controle de mesma idade. No entanto, os grupos foram categorizados pela massa corporal e não pelo TAV, e as associações com a VFC não foram comparadas entre os vários marcadores de composição corporal.

O músculo esquelético apoia a saúde metabólica e o funcionamento físico.^18^ A massa muscular expressa em relação ao TAV, indexada pela razão entre a classificação de massa muscular e gordura visceral (RMMCGC), parece ser um indicador relevante da função metabólica. Por exemplo, a RMMCGC derivado da BIA tem sido associada à doença hepática gordurosa não alcoólica e à fibrose hepática^19^ e auxilia na detecção da síndrome metabólica em adultos jovens.^20^ Assim, a RMMCGC parece ser um marcador de saúde relevante, mas a nossa compreensão da sua associação com o funcionamento autonómico cardíaco em adultos saudáveis é limitada.

O esclarecimento de como as características da composição corporal derivadas da BIA influenciam a VFC pode ser de relevância clínica para avaliar o risco e orientar o tratamento. Portanto, o objetivo desta investigação foi testar a hipótese de que a VFC difere entre os participantes em função da categorização do TAV e que os índices de TAV estarão mais fortemente associados à VFC do que a massa gorda relativa em homens adultos saudáveis.

## Métodos

### Configurações de estudo

Este estudo transversal foi realizado nos laboratórios do Departamento de Fisiologia e Fisiologia do Exercício, da Faculdade de Ciências do Esporte e Atividade Física da Universidade King Saud (KSU). Noventa e nove indivíduos sauditas adultos saudáveis do sexo masculino com idades entre 20 e 60 anos foram elegíveis para participação. A amostragem de conveniência foi utilizada para recrutar voluntários que foram abordados através de quadros de avisos, cartazes e anúncios nas redes sociais e aqueles que manifestaram interesse em participar foram informados de todos os procedimentos do estudo. Os critérios de inclusão foram: homens saudáveis, não fumantes; aqueles livres de distúrbios metabólicos, ortopédicos e neurológicos; sem história prévia de angina ou infarto do miocárdio e IMC < 40 kg/m^2^. Atletas profissionais foram excluídos, mas indivíduos altamente ativos recreativamente foram incluídos. Este estudo foi aprovado pelo Conselho de Revisão Institucional da KSU, Riad, Arábia Saudita (IRB No. E-18-3381). Todos os sujeitos forneceram consentimento informado por escrito e todos os procedimentos foram realizados de acordo com as diretrizes e protocolos relevantes. Os principais dados do artigo estão resumidos na Figura Central .

**Figure:**
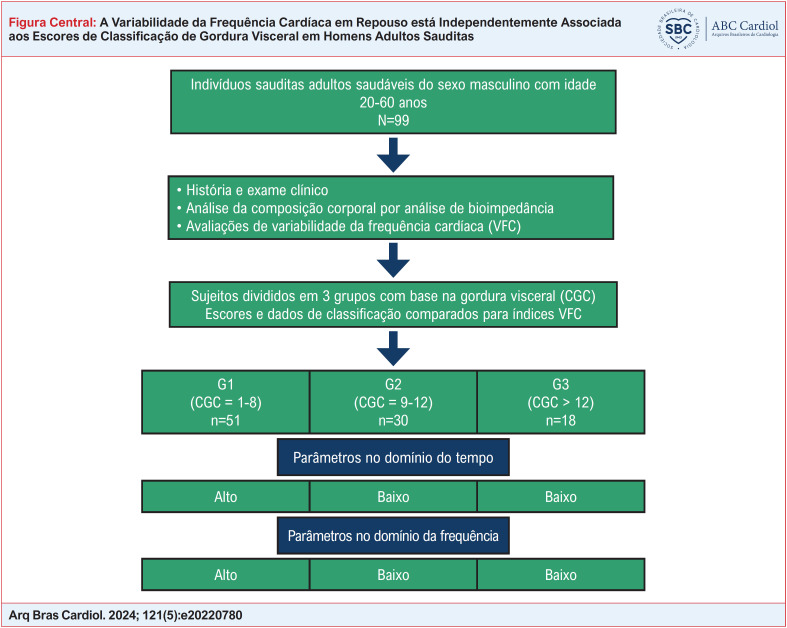


### Procedimentos de estudo

Os participantes foram instruídos a evitar a ingestão de bebidas com cafeína e exercícios vigorosos no dia anterior à visita ao laboratório para avaliação matinal em jejum noturno. As bebidas matinais, incluindo água, foram restritas para controle de sua influência confusa na VFC^21^ e parâmetros BIA.^22^ Todas as medições foram realizadas com procedimentos padrão que incluíam pressão arterial, dados demográficos, composição corporal e avaliação de eletrocardiograma (ECG).

### Pressão arterial e medidas antropométricas

A altura foi medida com um estadiômetro (Seca 213, Seca GmbH & Co., Hamburgo, Alemanha) e o peso corporal foi medido com uma balança digital (PD100 ProDoc, Detecto Scale, Cardinal, Webb City, MO, EUA). A circunferência da cintura (CC) foi medida em cm na altura do umbigo. Todos os valores foram ajustados para o 0,1 cm mais próximo. O índice de massa corporal (IMC) foi calculado dividindo-se o peso pela altura em metros quadrados. A frequência cardíaca de repouso e as pressões arteriais sistólica e diastólica foram estimadas por um esfigmomanômetro digital automatizado (HEM-7121, Omron, Shimogyo-ku, Kyoto, Japão).

### Estimativa de composição corporal

Uma máquina segmentar multifrequencial (MC-980MA, Tanita Corporation, Tóquio, Japão) que fornece correntes de 50-1000 kHz foi usada para estimar os parâmetros de composição corporal. Os participantes permaneceram descalços na balança enquanto seguravam os eletrodos com leve abdução glenoumeral. A altura de cada sujeito foi medida e registrada. Posteriormente, os participantes foram pesados e os valores de composição corporal foram estimados indiretamente por meio do aparelho. Foram coletadas as seguintes medidas de composição corporal: percentual de gordura corporal (GC%), percentual de água corporal, massa muscular, massa óssea e classificação de gordura visceral (CGC).^14^ A CGC é fornecida como uma classificação específica (0-59 pontos). Avaliações de 1 a 12 pontos indicam que o sujeito possui nível de gordura visceral saudável, enquanto classificações de 13 a 59 indicam que o sujeito possui nível de gordura visceral em excesso.^23 - 25^ A CGC tem sido amplamente aplicada em pesquisas clínicas como um índice indireto de gordura visceral. A RMMCGC foi posteriormente calculada.^26^

### Avaliação da variabilidade da frequência cardíaca

Todos os participantes foram submetidos a testes de ECG padrão de 12 derivações na posição supina após a avaliação da composição corporal. Utilizamos um dispositivo computadorizado de aquisição de dados de ECG (PL3516 Power Lab 16/35, AD Instruments Pty Ltd. New South Wales, Austrália) com 16 canais de entrada analógica. Um registro de ECG de 10 minutos foi realizado enquanto a qualidade do sinal era monitorada por um pesquisador. Um software customizado (LabChart v. 8.1.13 Windows, AD Instruments Pty Ltd. New South Wales, Austrália) foi utilizado para calcular as variáveis de VFC. Artefatos e batimentos ectópicos foram processados usando um filtro padronizado pela ferramenta "Beat Classification" automatizada por software, que categoriza os batimentos de acordo com atividade e ruído isoelétrico e remove artefatos gerados por movimento, interferência elétrica e batimentos ectópicos. Os parâmetros da VFC foram derivados de um segmento critério de 5 minutos (ou seja, 5-10 minutos) e os primeiros 5 minutos foram descartados para estabilização.^27^ Os parâmetros no domínio do tempo registrados para análise foram o tempo médio entre os intervalos RR (RR médio), o desvio padrão dos intervalos RR normais (SDNN) e a raiz quadrada da média das diferenças sucessivas das diferenças normais dos intervalos RR (RMSSD). A análise no domínio da frequência da VFC pelo método da transformada rápida de Fourier incluiu avaliação de baixa frequência (BF), alta frequência (AF) e baixa relação BF/AF. O RR médio reflete a frequência cardíaca em repouso, o RMSSD e o AF refletem a modulação parassimpática, o SDNN e o BF refletem a variabilidade global com influência simpática e parassimpática.^5^

### Análise estatística

Para entrada de dados e análise estatística foi utilizado o software SPSS (versão 20.0 Chicago, IL, EUA). As variáveis contínuas foram apresentadas como média e desvio padrão (DP) enquanto as variáveis categóricas foram expressas como frequências e/ou porcentagens (%). As variáveis contínuas foram verificadas quanto à normalidade por meio do teste de Kolmogorov-Smirnov. Os dados que seguem distribuições não normais foram transformados em log. Os participantes foram categorizados em 3 grupos (G) de acordo com a CGC: G1 (CGC = 1-8), G2 (CGC = 9-12) e G3 (CGC > 12). As características da VFC e da gordura corporal estão associadas à idade, o que pode confundir as associações entre a VFC e as características da gordura corporal. Assim, os grupos estratificados por CGC foram comparados quanto às características demográficas e parâmetros de VFC por meio de análise de covariância unidirecional (ANCOVA) com ajuste para idade e todas as suposições necessárias para o uso da ANCOVA foram atendidas. O teste do qui-quadrado foi utilizado para comparar as distribuições percentuais entre os diferentes grupos. Comparações post-hoc foram realizadas utilizando os testes de Bonferroni e Tamhane. Associações bivariadas entre VFC bruta e parâmetros de composição corporal foram avaliadas com o ρ de Spearman. Finalmente, foram realizadas regressões lineares múltiplas separadas com %GC, CGC e RMMCGC como preditores para avaliar o valor preditivo comparativo de cada um com os parâmetros da VFC. Os modelos incluíram idade, IMC e pressão arterial sistólica e diastólica como covariáveis. P<0,05 foi considerado estatisticamente significativo.

## Resultados

Os valores demográficos para todo o grupo (n = 99) e grupos estratificados por CGC são apresentados na Tabela 1 . O G1 era significativamente mais jovem que o G2 e o G3. Após controle por idade, o G1 apresentou massa corporal, massa muscular e circunferência da cintura significativamente menores, além de características de gordura corporal mais baixas do que G2 e G3. Além disso, o G3 apresentou circunferência da cintura ajustada por idade e características de gordura corporal significativamente maiores que o G2.

**Tabela 1:** Dados demográficos e análise da composição corporal de todos os indivíduos e subgrupos com base na classificação de gordura visceral

Variáveis	Todos	G 1 (CGC = 1-8)	G 2 (CGC = 9-12)	G 3 (CGC > 12)	Valor p	Valor de p ajustado por idade
N	99	51	30	18		
CGC	8,91±5,21	5,02±2,75	11,07±1,39	16,33±4,35	0,000	0,000
Idade (ano)	37,87±13,46	32,02±10,67	42,53±11,53	46,67±16,13	0,000	-
Altura (cm)	171,94±6,46	171,84±5,99	173,10±7,44	170,28±5,95	0,342	0,468
Peso (kg)	78,98±15,72	71,36±10,05 a . C .	87,39±11,67	86,55±23,13	0,000	0,000
IMC (kg/m^2^)	26,82±4,94	23,87 4,01b c	28.02 3.19	31,69 4,32	0,000	0,000
CC (cm)	100,63±8,54	80,62±8,03 a . C .	92,73±7,59	100,63±8,54 b	0,000	0,000
PAS (mmHg)	121,59±16,14	110,00±13,80 a . C .	119,13±18,18	119,13±18,18	0,008	0,447
PAD (mmHg)	74,86±10,68	72,10±10,33	76,53±12,07	80,00 ± 6,07 a	0,847	0,573
Porcentagem de gordura (%)	22,86±6,31	18,17±4,40 a . C .	26,45±3,27	30,17 ± 2,98 b	0,000	0,000
Massa gorda (kg)	19,15±8,19	13h30 ± 4,66 a . C .	23,60±5,44	28,29±6,79b	0,000	0,000
MLG (kg)	60,82±7,73	57,89±6,49 a . C .	64,09±7,48	63,71±8,48	0,000	0,001
Massa muscular (kg)	57,79±7,41	55,15±6,27 a . C .	60,57±7,30	60,64±8,14	0,001	0,001
Razão RMMCGC	9,66±7,27	14,15±7,77 a . C.	5,52±0,70	3,88±0,85	0,000	0,000
SM n(%)						
Sim	27(27,27)	13(14,94)	8(8,79)	8(5,27)	0,293	
Não	72(72,72)	38(36,06)	22(21,21)	10(12,73)		

A diferente de G1;

b diferente de G2;

c diferente de G3;

Os dados são apresentados como média ± DP. G: grupo; CGC: classificação de gordura visceral; N: número de participantes; IMC: índice de massa corporal; CC: circunferência da cintura; PAS: pressão arterial sistólica; PAD: pressão arterial diastólica; MLG: massa livre de gordura; RMMCGC: relação entre massa muscular e gordura visceral; SM: síndrome metabólica. A análise unidirecional de covariância (ANCOVA) foi usada para comparações de grupos. As proporções foram comparadas pelo Qui-quadrado.

A comparação da VFC no domínio do tempo revelou diferenças significativas entre grupos para RMSSD e SDNN, mas não para RR médio ( Tabela 2 ). Análises post-hoc demonstraram que RMSSD e SDNN foram significativamente maiores no G1 do que no G2 e G3, independentemente da idade. A comparação da VFC no domínio da frequência revelou diferenças significativas entre os grupos para BF, mas não para AF ou BF/AF ( Tabela 3 ). Análises post-hoc determinaram que a BF foi significativamente maior no G1 do que no G2, independentemente da idade.

**Tabela 2:** Análise no domínio do tempo de todos os indivíduos e subgrupos com base na classificação da gordura visceral

Variáveis	Todos	G 1 (CGC = 1-8)	G 2 (CGC = 9-12)	G 3 (CGC > 12)	Valor p	Valor p ajustado
N	99	51	30	18		
FC média (bpm)	59,86±6,06	58,89±5,37	59,96±6,42	62,47±6,84	0,098	0,122
RR médio (s)	1,02±0,10	1,03±0,09	1,01±0,10	0,97±0,11	0,090	0,117
SDNN (ms)	1.664±0,258	1.776±0,229^b,c^	1.543±0,258	1.548±0,193	0,000	0,007
RMSSD (ms)	1.666±0,258	1.779±0,225^b,c^	1.543±0,259	1.553±0,202	0,000	0,006

Os dados são apresentados como média ± DP; G: grupo; CGC: classificação de gordura visceral; N: número de participantes; RR médio: tempo médio entre os complexos QRS (ou seja, batimento a batimento); SDNN: desvio padrão dos intervalos RR normais; RMSSD: raiz quadrada da média das diferenças sucessivas dos intervalos RR normais. A análise unidirecional de covariância (ANCOVA) foi usada para comparações de grupos.

**Tabela 3:** Análise dos domínios de frequência de todos os indivíduos e subgrupos com base na classificação da gordura visceral

Variáveis	Todos	G 1 (CGC = 1-8)	G 2 (CGC = 9-12)	G 3 (CGC > 12)	Valor p	Valor p ajustado
N	99	51	30	18		
BF (ms^2^)	2.884±0,599	3.106±0,459b	2.620±0,732	2.766±0,445	0,007	0,027
AF (ms^2^)	2.928±0,674	3.229±0,459	2.584±0,828	2.766±0,514	0,004	0,284
BF/AF	-0,130±0,330	-0,190±0,347	-0,155±0,231	0,081±0,349b	0,001	0,496

Os dados são apresentados como média ± DP; G: grupo; CGC: classificação de gordura visceral; N: número de participantes; BF: baixa frequência; AF: alta frequência. A análise unidirecional de covariância (ANCOVA) foi usada para comparações de grupos.

**Figura 1:**
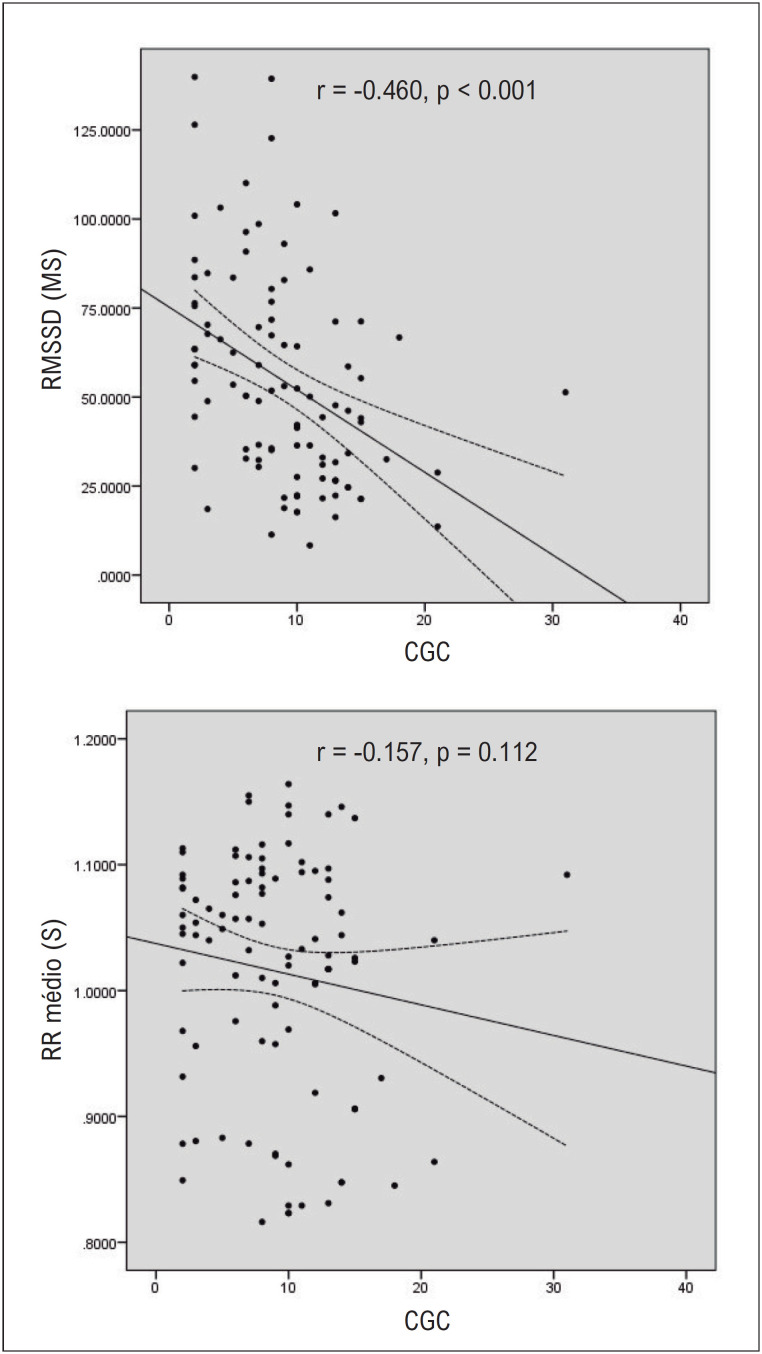
Gráficos de dispersão das relações CGC com RMSSD e RR médio. CGC: classificação de gordura corporal; RMSSD: raiz quadrada média de diferenças sucessivas.

Gráficos de dispersão comparando associações entre RMSSD e RR médio com CGC são apresentados na Figura 1 .

Após ajuste de covariáveis nas análises de regressão, a CGC foi significativamente preditiva de RMSSD, SDNN e AF, e o RMMCGC foi significativamente preditivo de RMSSD e SDNN. Os coeficientes Ɓeta padronizados são apresentados na Tabela 4 .

**Tabela 4:** Análise de regressão linear múltipla com %GC, CGC e RMMCGC como preditores, ajustando para covariáveis idade, massa corporal e pressão arterial sistólica e diastólica

Variável Dependente em Modelos de Regressão		Coeficientes Padronizados Beta	t	Valor p
RR médio (S)	%GC	-.303	-1,865	0,065
	CGC	-.083	-.496	0,621
	RMMCGC	-.007	-.042	0,966
SDNN (ms)	%GC	-.197	-1.307	.194
	CGC	-.421	-3.206	0,002
	RMMCGC	0,283	2.372	0,020
RMSSD (ms)	%GC	-.184	-1.221	0,225
	CGC	-.410	-3.117	0,002
	RMMCGC	0,275	2.305	0,023
BF (ms^2^)	%GC	-.032	-.205	0,838
	CGC	-.167	-1.038	.302
	RMMCGC	-.067	-.432	0,667
AF (ms^2^)	%GC	-.089	-.557	0,579
	CGC	-.317	-2.248	0,027
	RMMCGC	.219	1.723	0,089
BF/AF	%GC	0,044	0,258	0,797
	CGC	0,083	0,477	0,635
	RMMCGC	0,120	0,702	0,484

RR médio: tempo médio entre os complexos QRS (ou seja, batimento a batimento); SDNN: desvio padrão dos intervalos RR normais; RMSSD: raiz quadrada da média das diferenças sucessivas dos intervalos RR normais; B: baixa frequência; AF: alta frequência.

## Discussão

Os objetivos desta investigação foram 1) comparar a VFC entre grupos estratificados por CGC derivado da BIA e 2) comparar associações entre a VFC e as métricas de composição corporal em homens sauditas saudáveis. As principais descobertas foram que os indivíduos na categoria de CGC mais baixa apresentaram RMSSD, SDNN e BF ajustados por idade significativamente mais altos do que grupos de categorização de CGC mais alta. Além disso, os parâmetros da VFC (RMSSD, SDNN, AF) foram mais fortemente associados ao CGC do que à %GC e à RMMCGC após controle para idade, IMC e pressão arterial sistólica e diastólica.

Em apoio à nossa hipótese, as métricas de influência vagal da VFC (RMSSD) e a variabilidade global (SDNN, BF) variaram em função da categorização da CGC derivada da BIA em uma amostra de homens saudáveis. Uma investigação anterior envolvendo adultos jovens saudáveis comparou parâmetros de composição corporal derivados da VFC e da BIA entre grupos categorizados pelo IMC.^28^ AF, BF e potência total normalizados significativamente mais elevados, e menor gordura corporal relativa e TAV, foram observados entre grupos de homens e mulheres categorizados como com sobrepeso (n = 40, IMC = 23,00 – 27,40) versus controles com peso normal (n = 40, IMC = 18,50 – 22,90).^28^ Além disso, foram relatadas associações bivariadas moderadas significativas entre TAV relativo e AF normalizada (r = −0,32) e BF (0,40).^28^ Nossas descobertas revelaram diferenças semelhantes entre grupos e magnitudes de correlação. Nossa inclusão de parâmetros de VFC no domínio do tempo mais comuns e acessíveis e ajuste para fatores de confusão em análises multivariadas acrescentam novos insights à literatura sobre a associação entre TAV e VFC em uma população saudável. Além disso, nosso achado de não haver diferença no RR médio entre os grupos CGC indica maior sensibilidade dos parâmetros da VFC ao acúmulo de TAV.

Foram recentemente relatadas associações transversais entre parâmetros de VFC e indicadores de TAV. Uma investigação envolvendo mulheres adultas jovens (n = 104) descobriu que o TAV derivado da absorciometria de raios X de dupla energia expresso em relação à massa corporal explicou ∼10-16% da variância no tempo (SDNN e RMSSD) e no domínio da frequência (AF e parâmetros BF)^29^ após ajuste de idade. Outra investigação envolvendo homens e mulheres multiétnicos mais jovens e mais velhos (n = 178) com e sem morbidades descobriu que maior circunferência da cintura estava associada à diminuição da VFC (RMSSD e SDNN derivado de Holter) em adultos mais jovens, mas não em idosos, independentemente de condições de saúde conhecidas e atividade física autorrelatada.^14^ Yi et al. descobriram que uma maior relação cintura-quadril estava associada à redução da VFC (por exemplo, RMSSD e BF) em um subgrupo de adultos coreanos com excesso de peso, independentemente da idade, sexo e fatores de risco cardiovascular. De acordo com os achados atuais, foi relatado que a gordura abdominal foi mais preditiva da VFC do que o percentual de gordura corporal derivado da BIA.^15^

A obesidade abdominal está associada a maior ativação simpática em repouso do que a obesidade periférica, apesar do comprometimento barorreflexo semelhante entre grupos obesos, implicando mecanismos de origem metabólica.^30^ Hiperglicemia, hiperinsulinemia e resistência à insulina estão associadas tanto à obesidade abdominal quanto à hiperatividade simpática, levando à redução da VFC e ao aumento do risco cardiometabólico.^31^ Entretanto, anormalidades metabólicas descritas acima estão ligadas a estados pró-inflamatórios.^32^ O TAV contribui para a inflamação sistêmica por meio da secreção de adipocinas, como fator de necrose tumoral-α, interleucina-6, proteína C reativa e angiotensinogênio.^33 , 34^ Foi demonstrado que a inflamação de baixo grau está associada à VFC atenuada em adultos de meia-idade e idosos.^35^ Além disso, acredita-se que o estresse oxidativo causado pela desregulação de espécies reativas de oxigênio relacionada à adipocina também contribua para o desequilíbrio autonômico.^31^ Assim, o TAV pode promover disfunção do SNA através dos efeitos de suas secreções, mas são necessários mais estudos visando esclarecer os mecanismos.

As limitações do nosso estudo incluem seu desenho transversal, utilização apenas de registros de VFC em posição supina e falta de covariáveis adicionais importantes, como biomarcadores sanguíneos^31 , 32^ e atividade física.^34^ A falta de controle respiratório durante a avaliação do ECG também pode ser considerada uma limitação. Além disso, o tamanho relativamente pequeno da nossa amostra e a inclusão apenas de homens limitam a generalização dos nossos resultados para a população em geral. Finalmente, o TAV foi estimado pela BIA, que é de qualidade inferior em relação a abordagens de critérios como a ressonância magnética.

## Conclusões e recomendações

Maior gordura visceral, conforme indexado pela CGC derivada da BIA, foi associada à redução da VFC em uma amostra de homens saudáveis. As associações permaneceram estatisticamente significativas após a idade, o IMC e a pressão arterial sistólica e diastólica foram mantidos constantes. A CGC foi mais fortemente associada à VFC quando comparada ao percentual de gordura corporal e à RMMCGC. O TAV e a VFC são modificáveis com fatores de estilo de vida e podem ser automonitorados com dispositivos disponíveis comercialmente. Nossas descobertas sugerem que o RMSSD e o SDNN podem ser os parâmetros de VFC mais sensíveis às mudanças no TAV e, portanto, devem ser os principais parâmetros de interesse no rastreamento do estado autonômico cardíaco em resposta a intervenções que visam a redução do TAV.
