# Supplementary material for: A Variabilidade da Frequência Cardíaca em Repouso está Independentemente Associada aos Escores de Classificação de Gordura Visceral em Homens Adultos Sauditas
Source: Arq Bras Cardiol. 2024 May 7;121(5):e20220780. [Article in Portuguese] doi: 10.36660/abc.20220780 (PMC11081131; doi:10.36660/abc.20220780)
Supplement: Translation

# Translation: Resting Heart Rate Variability is Independently Associated with Visceral Fat Rating Scores in Saudi Adult Males

**DOI:** 10.36660/abc.20220780i

**Authors:** Syed Shahid Habib, Shaea Alkahtani, Nouf Aljawini, Syed Mohammad Habib, Andrew A. Flatt

**Affiliations:** 1 Riyadh Saudi Arabia Department of Physiology, College of Medicine, King Saud University, Riyadh – Saudi Arabia; 2 Riyadh Saudi Arabia Department of Exercise Physiology, College of Sport Sciences and Physical Activity, King Saud University, Riyadh – Saudi Arabia; 3 Riyadh Saudi Arabia Department of Community Health Sciences, College of Applied Medical Sciences, King Saud University, Riyadh – Saudi Arabia; 4 Al Bukairiyah Saudi Arabia College of Medicine, Sulaiman Al Rajhi Colleges, Al Bukairiyah – Saudi Arabia; 5 Savannah USA Department of Health Sciences and Kinesiology, Biodynamics and Human Performance Center, Georgia Southern University-Armstrong, Savannah – USA

**Keywords:** Intra-Abdominal Fat, Heart Rate, Autonomic Nervous System

## Abstract

**Background:**

Visceral adipose tissue (VAT) may be a specific modifiable contributor to body composition-related autonomic impairment.

**Objectives:**

To compare heart rate variability (HRV) between groups stratified by visceral fat rating (VFR) and compare associations between HRV and body composition metrics.

**Methods:**

A cross-sectional study was conducted on healthy men (n=99,age=37.8±13.4 years, body mass index [BMI]=26.9±4.6 kg/m^2^). HRV was derived from 5-minute electrocardiographic recordings. Body composition (body fat percentage, VFR, and muscle mass to visceral fat ratio [MMVFR]) was estimated using tetrapolar bioelectrical impedance analysis. Participants were categorized into groups according to VFR: G1 (VFR=1-8); G2(VFR=9-12); and G3(VFR>12). Age-adjusted comparisons were made between groups. Independent associations were quantified with multiple linear regressions. P <0.05 was significant.

**Results:**

Root-mean square of successive differences (RMSSD) and standard deviation of normal RR intervals (SDNN) were higher for G1 vs. G2 and G3 (p<0.05). Low-frequency power (LF) was higher in G1 than in G2 (p<0.05). VFR and MMVFR were negatively associated with SDNN, RMSSD, LF, and HF (p<0.05). After adjusting for age, BMI, and systolic and diastolic blood pressure, VFR was significantly predictive of RMSSD, SDNN, and HF (p=0.002,–0.027), and MMVFR was significantly predictive of RMSSD and SDNN (p=0.020,–0.023).

**Conclusions:**

Men in the lowest VFR category had the highest HRV. VFR was more strongly associated with HRV than body fat percentage and MMVR. Time domain parameters were more sensitive to VAT than frequency domain parameters. HRV parameters could be the primary parameters of interest in tracking cardiac-autonomic status in response to interventions targeting VAT reduction.

## Introduction

Cardiovascular disease (CVD) continues to be a global health burden as a leading cause of morbidity and mortality.^1^A growing body of evidence demonstrates an association between increased adiposity and autonomic nervous system (ANS) imbalance,^2 - 4^inferred from heart rate variability (HRV) analysis. HRV reflects centrally-mediated cardiac control via autonomic innervation.^5^Vagal influence increases HRV whereas attenuated vagal functioning, or increased sympathetic stimulation, reduces HRV.^6^Cardiac-parasympathetic hypoactivity is associated with an elevated risk of incident coronary heart disease and all-cause mortality.^7^However, HRV is modifiable with lifestyle modification and appears to be sensitive to changes in body composition.^8^

Though high body and fat mass have been associated with reduced HRV in a variety of young,^9^healthy,^10^athletic,^11^and clinical populations,^12^site-specific fat deposition is unaccounted for in these general body composition markers. Visceral adipose tissue (VAT) that surrounds vital organs within the abdomen is more hazardous to human health than subcutaneous fat and is more predictive of cardiometabolic risk.^13^Among obese individuals, those with less VAT storage typically exhibit a more favorable risk profile than those with higher VAT. A limited number of investigations have compared associations between HRV and markers of total body fat and central fat storage. Studies in women, elderly, and a Korean population have reported stronger associations between HRV and indicators of VAT.^14 - 16^Thus, central adiposity seems to be influencing the association between body fat and cardiac-autonomic function.

VAT can be estimated with tetrapolar bioelectrical impedance analysis (BIA), which serves as a convenient alternative to criterion laboratory measures or basic anthropometry.^17^To our knowledge, only one previous investigation has quantified associations between BIA-derived VAT and HRV in healthy adults. Results showed lower vagal-mediated HRV in overweight healthcare students versus an age-matched control group. However, groups were categorized by body mass rather than by VAT, and associations with HRV were not compared between the various markers of body composition.

Skeletal muscle supports metabolic health and physical functioning.^18^Muscle mass expressed relative to VAT, indexed by the ratio of muscle mass to visceral fat ratio (MMVFR), seems to be a relevant indicator of metabolic function. For example, BIA-derived MMVFR has been associated with non-alcoholic fatty liver disease and liver fibrosis^19^and aids in the detection of Metabolic Syndrome in young adults.^20^Thus, MMVFR seems to be a relevant health marker, but our understanding of its association with cardiac-autonomic functioning in healthy adults is limited.

Clarification of how BIA-derived body composition characteristics influence HRV may be of clinical relevance for assessing risk and guiding treatment. Therefore, the purpose of this investigation was to test the hypothesis that HRV differs between participants as a function of VAT categorization and that indexes of VAT will be more strongly associated with HRV than relative fat mass in healthy adult men.

## Methods

### Study settings

This cross-sectional study was conducted at the Department of Physiology and Exercise Physiology laboratories, within the College of Sport Sciences and Physical Activity of King Saud University (KSU). Ninety-nine healthy adult Saudi male subjects aged 20-60 years were eligible for participation. Convenience sampling was used to recruit volunteers who were approached via bulletin boards, posters, and social media announcements and those who expressed interest in participating were informed of all study procedures. Inclusion criteria included: healthy men non-smokers; those free of metabolic, orthopedic, and neurological disorders; no history of previous angina or myocardial infarction and BMI < 40 kg/m^2^. Professional athletes were excluded, but highly recreationally active individuals were included. This study was approved by the Institutional Review Board of KSU, Riyadh, Saudi Arabia (IRB No. E-18-3381). All subjects provided written informed consent and all procedures were performed as per the relevant guidelines and protocols. The main data of the article is summarized in the Central Illustration .

### Study procedures

Participants were instructed to avoid intake of caffeinated drinks and vigorous exercise training on the day before the laboratory visit for morning assessment in an overnight fasted state. Morning beverages, including water, were restricted to control for their confounding influence on HRV^21^and BIA^22^parameters. All measurements were performed with standard procedures that comprised blood pressure, demographics, body composition, and electrocardiogram (ECG) assessment.

### Blood pressure and anthropometric measurements

Height was measured using a stadiometer (Seca 213, Seca GmbH & Co., Hamburg, Germany), and body weight was measured with a digital scale (PD100 ProDoc, Detecto Scale, Cardinal, Webb City, MO, USA). Waist circumference (WC) was measured in cm at the umbilicus. All values were adjusted to the nearest 0.1 cm. Body mass index (BMI) was computed by dividing weight by height in squared meters. Resting heart rate and systolic and diastolic blood pressures were estimated by an automated digital sphygmomanometer (HEM-7121, Omron, Shimogyo-ku, Kyoto, Japan).

### Body composition estimation

A multi-frequency segmental machine (MC-980MA, Tanita Corporation, Tokyo, Japan) that delivers 50-1000 kHz currents was used to estimate body composition parameters. Participants stood barefooted on the scale whilst holding the electrodes with slight glenohumeral abduction. The height of each subject was measured and recorded. Participants were subsequently weighed and body composition values were indirectly estimated using the device. The following body composition measures were collected: body fat percentage (BF%), body water percentage, muscle mass, bone mass, and visceral fat rating (VFR).^14^VFR is provided as a specific rating (0–59 points). Ratings from 1 to 12 points indicate that the subject has a healthy visceral fat level, while ratings from 13 to 59 indicate that the subject has an excess visceral fat level.^23 - 25^VFR has been widely applied in clinical research as an indirect index of visceral fat. The MMVR was subsequently computed.^26^

### Heart rate variability assessment

All participants underwent 12-lead standard ECG testing in the supine position following the body composition assessment. We used a computerized ECG data acquisition device (PL3516 Power Lab 16/35, AD Instruments Pty Ltd. New South Wales, Australia) with 16 analog input channels. A 10-minute ECG recording was performed while signal quality was monitored by a researcher. A customized software (LabChart v. 8.1.13 Windows, AD Instruments Pty Ltd. New South Wales, Australia) was used for computing HRV variables. Artifacts and ectopic beats were processed using a standardized filter by the software-automated "Beat Classification" tool, which categorizes beats according to activity and isoelectric noise, and removes artifacts generated by movement, electrical interference, and ectopic beats. HRV parameters were derived from a 5-min criterion segment (i.e., min 5-10) and the first 5-min were discarded for stabilization.^27^Time-domain parameters recorded for analysis were the average time between RR intervals (Average RR), the standard deviation of normal RR intervals (SDNN), and the root mean square of the successive differences of normal RR interval differences (RMSSD). Frequency domain analysis of HRV by fast Fourier transform method included assessment of low frequency (LF), high frequency (HF), and the low LF/HF ratio. Average RR reflects resting heart rate, RMSSD and HF reflect parasympathetic modulation, SDNN, and LF reflect global variability with sympathetic and parasympathetic influence.^5^

### Statistical analysis

For data entry and statistical analysis, SPSS software (version 20.0 Chicago, IL, USA) was used. Continuous variables were presented as mean and standard deviation (SD) while categorical variables were expressed as frequencies and/or percentages (%). Continuous variables were checked for normality using the Kolmogorov-Smirnov test. The data following non-normal distributions were log-transformed. Participants were categorized into 3 groups (G) according to their VFR: G1 (VFR = 1-8), G2 (VFR = 9-12), and G3 (VFR > 12). HRV and body fat characteristics are both associated with age, which may confound associations between HRV and body fat characteristics. Thus, groups stratified by VFR were compared for demographic characteristics and HRV parameters by one-way analysis of covariance (ANCOVA) adjusting for age and all the assumptions necessary for the use of ANCOVA were met. The chi-square test was used to compare percentage distributions between different groups. Post-hoc comparisons were performed using Bonferroni and Tamhane tests. Bivariate associations between raw HRV and body composition parameters were assessed with Spearman's ρ. Finally, separate multiple linear regressions were performed with BF%, VFR, and MMVFR as predictors to assess the comparative predictive value of each with HRV parameters. Models included age, BMI, and systolic and diastolic blood pressure as covariates. P <0.05 was considered statistically significant.

## Results

Demographic values for the entire group (n = 99) and groups stratified by VFR are displayed in Table 1 . G1 was significantly younger than G2 and G3. After controlling for age, G1 had significantly lower body mass, muscle mass, and waist circumference, along with lower body fat characteristics than G2 and G3. In addition, G3 had significantly higher age-adjusted waist circumference and body fat characteristics than G2.

**Table 1:** Demographic data and body composition analysis of all subjects and subgroups based on visceral fat rating

Variables	All	G 1 (VFR = 1-8)	G 2 (VFR = 9-12)	G 3 (VFR > 12)	p value	p value adjusted for age
N	99	51	30	18		
VFR	8.91 ± 5.21	5.02 ± 2.75	11.07 ± 1.39	16.33 ± 4.35	.000	.000
Age (year)	37.87 ± 13.46	32.02 ± 10.67	42.53 ± 11.53	46.67 ± 16.13	.000	-
Height (cm)	171.94 ± 6.46	171.84 ± 5.99	173.10 ± 7.44	170.28 ± 5.95	.342	.468
Weight (kg)	78.98 ± 15.72	71.36 ± 10.05 b c	87.39 ± 11.67	86.55 ± 23.13	.000	.000
BMI (kg/m^2^)	26.82 ± 4.94	23.87 4.01 b c	28.02 3.19	31.69 4.32	.000	.000
WC (cm)	100.63 ± 8.54	80.62 ± 8.03 b c	92.73 ± 7.59	100.63 ± 8.54 b	.000	.000
SBP (mmHg)	121.59 ± 16.14	110.00 ± 13.80 b c	119.13 ± 18.18	119.13 ± 18.18	.008	.447
DBP (mmHg)	74.86 ± 10.68	72.10 ± 10.33	76.53 ± 12.07	80.00 ± 6.07 a	.847	.573
Fat percentage (%)	22.86 ± 6.31	18.17 ± 4.40 b c	26.45 ± 3.27	30.17 ± 2.98 b	.000	.000
Fat mass (kg)	19.15 ± 8.19	13.30 ± 4.66 b c	23.60 ± 5.44	28.29 ± 6.79 b	.000	.000
FFM (kg)	60.82 ± 7.73	57.89 ± 6.49 b c	64.09 ± 7.48	63.71 ± 8.48	.000	.001
Muscle mass (kg)	57.79 ± 7.41	55.15 ± 6.27 b c	60.57 ± 7.30	60.64 ± 8.14	.001	.001
MMVFR	9.66 ± 7.27	14.15 ± 7.77 b c	5.52 ± 0.70	3.88 ± 0.85	.000	.000
MetS n(%)						
Yes	27(27.27)	13(14.94)	8(8.79)	8(5.27)	.293	
No	72(72.72)	38(36.06)	22(21.21)	10(12.73)		

A different from G1;

b different from G2;

c different from G3.

Data is presented as mean ± SD. G: group; VFR: visceral fat rating; N: number of participants; BMI: body mass index; WC: waist circumference; SBP: systolic blood pressure; DBP: diastolic blood pressure; FFM: fat-free mass; MMVFR: muscle mass to visceral fat ratio; MetS: metabolic syndrome. One-way analysis of covariance (ANCOVA) was used for group comparisons. Proportions were compared by Chi-square.

The time-domain HRV comparison revealed significant between-group differences for RMSSD and SDNN, but not Average RR ( Table 2 ). Post-hoc analyses demonstrated that RMSSD and SDNN were significantly higher for G1 than G2 and G3, independent of age. Comparison of frequency domain HRV revealed significant between-group differences for LF, but not HF or LF/HF ( Table 3 ). Post-hoc analyses determined that LF was significantly higher in G1 than in G2, independent of age.

**Table 2:** Time domain analysis of all subjects and subgroups based on visceral fat rating

Variables	All	G 1 (VFR = 1-8)	G 2 (VFR = 9-12)	G 3 (VFR > 12)	p value	p value adjusted
N	99	51	30	18		
Average HR (bpm)	59.86 ± 6.06	58.89 ± 5.37	59.96 ± 6.42	62.47 ± 6.84	0.098	0.122
Average RR (s)	1.02 ± 0.10	1.03 ± 0.09	1.01 ± 0.10	0.97 ± 0.11	0.090	0.117
SDNN (ms)	1.664± 0.258	1.776± 0.229^b c^	1.543± 0.258	1.548± 0.193	0.000	0.007
RMSSD (ms)	1.666 ± 0.258	1.779 ± 0.225^b c^	1.543± 0.259	1.553± 0.202	0.000	0.006

Data is presented as mean ± SD; G: group; VFR: visceral fat rating; N: number of participants; Average RR: the average time between QRS complexes (i.e. beat to beat); SDNN: standard deviation of normal RR intervals; RMSSD: Root mean square of the successive differences of normal RR intervals. One-way analysis of covariance (ANCOVA) was used for group comparisons.

**Table 3:** Frequency domains analysis of all subjects and subgroups based on visceral fat rating

Variables	All	G 1 (VFR = 1-8)	G 2 (VFR = 9-12)	G 3 (VFR > 12)	p value	p value adjusted
N	99	51	30	18		
LF (ms^2^)	2.884± 0.599	3.106± 0.459^b^	2.620± 0.732	2.766± 0.445	.007	.027
HF (ms^2^)	2.928± 0.674	3.229± 0.459	2.584± 0.828	2.766± 0.514	.004	.284
LF/HF	-0.130± 0.330	-0.190± 0.347	-0.155± 0.231	0.081± 0.349^b^	.001	.496

Data is presented as mean ± SD; G: group; VFR: visceral fat rating; N: number of participants; LF: low frequency; HF: high frequency. One-way analysis of covariance (ANCOVA) was used for group comparisons.

Scatterplots comparing associations between RMSSD and Average RR with VFR are presented in Figure 1 .

**Figure 1:** Scatter plots of VFR relationships with RMSSD and Average RR. VFR: visceral fat rating; RMSSD: Root-mean square of successive differences.

After covariate adjustment in regression analyses, VFR was significantly predictive of RMSSD, SDNN, and HF, and MMVFR was significantly predictive of RMSSD and SDNN. Standardized Ɓeta coefficients are presented in Table 4 .

**Table 4:** Multiple linear regression analysis with BF%, VFR, and MMVFR as predictors, adjusting for covariates of age, body mass, and systolic and diastolic blood pressure

Dependent Variable in Regression Models		Standardized Coefficients Beta	t	p value
Average RR (S)	BF%	-.303	-1.865	.065
	VFR	-.083	-.496	.621
	MMVFR	-.007	-.042	.966
SDNN (ms)	BF%	-.197	-1.307	.194
	VFR	-.421	-3.206	.002
	MMVFR	.283	2.372	.020
RMSSD (ms)	BF%	-.184	-1.221	.225
	VFR	-.410	-3.117	.002
	MMVFR	.275	2.305	.023
LF (ms^2^)	BF%	-.032	-.205	.838
	VFR	-.167	-1.038	.302
	MMVFR	-.067	-.432	.667
HF (ms^2^)	BF%	-.089	-.557	.579
	VFR	-.317	-2.248	.027
	MMVFR	.219	1.723	.089
LF/HF	BF%	.044	.258	.797
	VFR	.083	.477	.635
	MMVFR	.120	.702	.484

Average RR: the average time between QRS complexes (i.e. beat to beat); SDNN: standard deviation of normal RR intervals; RMSSD: Root mean square of the successive differences of normal RR intervals; LF: low frequency; HF: high frequency; MMVRF: muscle mass to visceral fat ratio; VFR: visceral fat rating.

## Discussion

The aims of this investigation were 1) to compare HRV between groups stratified by BIA-derived VFR and 2) to compare associations between HRV and body composition metrics in healthy Saudi men. The main findings were that individuals in the lowest VFR category had significantly higher age-adjusted RMSSD, SDNN, and LF than groups of higher VFR categorization. In addition, HRV parameters (RMSSD, SDNN, HF) were more strongly associated with VFR than BF% and MMVFR after controlling for age, BMI, and systolic and diastolic blood pressure.

In support of our hypothesis, HRV metrics of vagal influence (RMSSD) and global variability (SDNN, LF) varied as a function of BIA-derived VFR categorization in a sample of healthy men. A prior investigation involving healthy young adults compared HRV and BIA-derived body composition parameters among groups categorized by BMI.^28^Significantly higher normalized HF, LF, and total power, and lower relative body fat and VAT, were observed among groups of men and women categorized as overweight (n=40, BMI = 23.00 – 27.40) versus normal weight controls (n=40, BMI = 18.50 – 22.90).^28^Additionally, significant moderate bivariate associations were reported between relative VAT and normalized HF (r = −0.32) and LF (0.40).^28^Our findings revealed similar between-group differences and correlation magnitudes. Our inclusion of more common and accessible time-domain HRV parameters and adjustment for confounders in multivariate analyses add new insight to the literature regarding the association between VAT and HRV in a healthy population. Moreover, our finding of no difference in Average RR between VFR groups indicates greater sensitivity of HRV parameters to VAT accumulation.

Cross-sectional associations between HRV parameters and indicators of VAT have recently been reported. One investigation involving young adult women (n = 104) found that dual-energy x-ray absorptiometry-derived VAT expressed relative to body mass explained ∼10-16% of the variance in time (SDNN and RMSSD) and frequency domain (HF and LF) parameters^29^following age-adjustment. Another investigation involving younger and older multi-ethnic men and women (n = 178) with and without morbidities found that higher waist circumference was associated with decreasing HRV (Holter-derived RMSSD and SDNN) in younger, but not older adults, independent of known health conditions and self-reported physical activity.^14^Yi et al. found that a greater waist-to-hip ratio was associated with reduced HRV (e.g., RMSSD and LF) in an overweight sub-group of Korean adults, independent of age, sex, and cardiovascular risk factors. In agreement with the current findings, it was reported that abdominal fat was more predictive of HRV than BIA-derived body fat percentage.^15^

Abdominal obesity is associated with greater resting sympathetic activation than peripheral obesity, despite similar baroreflex impairment between obese groups, implying mechanisms of metabolic origin.^30^Hyperglycemia, hyperinsulinemia, and insulin resistance are associated with both abdominal obesity and sympathetic hyperactivity, leading to reduced HRV and increased cardiometabolic risk.^31^However, metabolic abnormalities as described above are linked to pro-inflammatory states.^32^VAT contributes to systemic inflammation via its secretion of adipokines such as tumor necrosis factor-α, interleukin-6, C-reactive protein, and angiotensinogen.^33 , 34^It's been demonstrated that low-grade inflammation is associated with attenuated HRV in middle-aged and older adults.^35^Moreover, oxidative stress from adipokine-related dysregulation of reactive oxygen species is also thought to contribute to autonomic imbalance.^31^Thus, VAT may promote dysfunction of the ANS via the effects of its secretions, but further study aimed at clarifying mechanisms is required.

Limitations of our study include its cross-sectional design, utilization of only supine HRV recordings, and lack of additional important covariates such as blood biomarkers^31 , 32^and physical activity.^34^Lack of respiratory control during ECG assessment may also be considered a limitation. Moreover, our relatively small sample size and inclusion of only men limit the generalizability of our findings to the broader population. Finally, VAT was estimated by BIA, which is of lower quality relative to criterion approaches such as magnetic resonance imaging.

## Conclusions and recommendations

Higher visceral fat, as indexed by BIA-derived VFR, was associated with reduced HRV in a sample of healthy men. Associations remained statistically significant after age, BMI and systolic and diastolic blood pressure were held constant. VFR was more strongly associated with HRV when compared to body fat percentage and MMVFR. VAT and HRV are modifiable with lifestyle factors and can be self-monitored with commercially available devices. Our findings suggest that RMSSD and SDNN may be the most sensitive HRV parameters to changes in VAT, and therefore should be the primary parameters of interest in tracking cardiac-autonomic status in response to interventions targeting VAT reduction.
